# The protective effects of orexin-A in alleviating cell senescence against interleukin-1β (IL-1β) in chondrocytes

**DOI:** 10.18632/aging.205884

**Published:** 2024-05-31

**Authors:** Lin Shen, Xiantie Zeng, Haiying Zhang

**Affiliations:** 1 Department of Orthopedics, Tianjin Hospital, Tianjin 300211, China; 2 Department of Orthopedics, Dongfang Hospital, Beijing University of Traditional Chinese Medicine, Beijing 100078, China

**Keywords:** osteoarthritis, orexin-A, cell senescence, IL-1β, SIRT3

## Abstract

Osteoarthritis (OA) is one of the most important causes of global disability, and dysfunction of chondrocytes is an important risk factor. The treatment of OA is still a challenge. Orexin-A is a hypothalamic peptide, and its effects in OA are unknown. In this study, we found that exposure to interleukin-1β (IL-1β) reduced the expression of orexin-2R, the receptor of orexin-A in TC-28a2 chondrocytes. Importantly, the senescence-associated β-galactosidase (SA-β-gal) staining assay demonstrated that orexin-A treatment ameliorates IL-1β-induced cellular senescence. Importantly, the presence of IL-1β significantly reduced the telomerase activity of TC-28a2 chondrocytes, which was rescued by orexin-A. We also found that orexin-A prevented IL-1β-induced increase in the levels of Acetyl-p53 and the expression of p21. It is shown that orexin-A mitigates IL-1β-induced reduction of sirtuin 3 (SIRT3). Silencing of SIRT3 abolished the protective effects of orexin-A against IL-1β-induced cellular senescence. These results imply that orexin-A might serve as a promising therapeutic agent for OA.

## INTRODUCTION

Osteoarthritis (OA) is a joint disease that is increasingly impacting society and the economy due to the accelerating aging of the population [1]. OA is caused by multiple risk factors, among which aging and obesity are the most prominent [2]. An epidemiological study in Europe showed that the incidence of OA gradually increases with age [3]. Data from the National Health Interview Survey in the United States also indicate that the incidence of symptomatic knee osteoarthritis (OA) peaks between the ages of 55 and 64, and the prevalence rate is positively correlated with age [4]. With the progression of OA, knee joint replacement surgery has been increasingly performed in late-stage OA to alleviate pain symptoms and disability status. However, even after joint replacement, over 60% of patients still experience long-term pain, which directly affects postoperative functional exercise and quality of life [5]. Understanding the role of aging in the occurrence and development of OA is of great significance for disease treatment and the development of new therapies. OA pathophysiology involves various joint tissue and cell types, with chondrocytes being a key focus in aging research. Chondrocytes are resident cells in articular cartilage, a highly differentiated avascular and a neural tissue whose structure and mechanical properties are primarily determined by extracellular matrix (ECM) components, including type II collagen and aggrecan produced by chondrocytes [6]. OA characteristics mainly include chondrocyte dysfunction and ECM degradation [7]. Chondrocytes control cartilage homeostasis, the senescence of which contributes to an imbalance between ECM synthesis and degradation, as well as reducing chondrocytes’ ability to maintain and restore articular cartilage [8]. Typical features of chondrocyte aging include enlarged cell size, decreased telomere length, overexpression of p21, p16, and p53 proteins, elevated levels of reactive oxygen species, and increased activity of senescence-associated β-galactosidase (SA-β-gal) [9]. A study by Martin et al. [10] has shown that SA-β-gal activity increases with age, while chondrocyte mitotic activity and telomere mean length decrease, leading to replicative senescence of chondrocytes *in vivo*. Regulating chondrocyte senescence may become an important treatment approach for OA. IL-1β is one of the central pro-inflammatory cytokines which has been involved in the progression of OA. Excessive production of IL-1β induces the expression of inflammatory mediators in human OA chondrocytes. Importantly, long-term exposure to IL-1β causes the senescence-associated secretory phenotype (SASP) of chondrocytes, which is an important pathological characteristic of OA [11].

Orexin, discovered independently in two separate laboratories in the United States in 1998 by Sakurai et al. and de Lecea et al., is a neuropeptide hormone synthesized and secreted by neurons in the lateral hypothalamus (LH). Orexin exists in two structurally different forms: orexin-A and orexin-B [12]. Initially, orexin-A was believed to primarily function in promoting feeding and energy metabolism. However, later studies revealed its critical role in regulating sleep homeostasis, the transition from sleep to wakefulness, and the maintenance of wakefulness [13, 14]. In recent years, researches have shown that orexin-A participates in the process of aging [15]. However, it remains unclear whether orexin-A has a regulatory effect on chondrocyte senescence and thereby affects the progression of OA. Our study aimed to explore the inhibitory effect of orexin-A on chondrocyte senescence and to clarify its potential therapeutic function for OA.

## MATERIALS AND METHODS

### Cell culture, treatment, and SIRT3 silencing

TC-28a2 chondrocytes were sourced from American Type Culture Collection (ATCC) (USA) and cultured in 90% Hyclone Dulbecco’s Modified Eagle’s Medium (DMEM) supplemented with high glucose and 10% fetal bovine serum (FBS), which were cultivated at 37°C and 5% CO_2_. To achieve SIRT3-silenced TC-28a2 chondrocytes, cells were transduced with adenovirus containing a shRNA targeting SIRT3 (Ad-SIRT3 shRNA) for 48 h and were identified using the Western blotting assay. Cells were stimulated with IL-1β (10 ng/ml) with or without orexin-A (5 and 10 μM).

### Real-time PCR assay

Total RNA was extracted from chondrocytes in each group after intervention using the TRIzol method (Invitrogen, USA). The content and purity of the RNA were checked with an ultra-micro spectrophotometer. The RNA was reversely transcribed into cDNA using a reverse transcription kit (QIAGEN, Germany), and the reaction system was prepared according to the manufacturer’s instructions, with 400 ng mRNA added to each reverse transcription system. The reaction procedure was as follows: 42°C for 15 min and 85°C for 5 min. Quantitative PCR was conducted using a qPCR kit (QIAGEN, Germany). The cDNA was diluted fivefold as the detection sample, and the progression was: pre-denaturation at 95°C for 10 min, denaturation at 95°C for 5 s, and annealing and extension at 60°C for 40 s (50 cycles). Glyceraldehyde-3-phosphate dehydrogenase (GAPDH) was taken as the confidential reference item, and the corresponding gene levels were analyzed using the 2^−ΔΔCt^ method.

### Enzyme-linked immunosorbent assay (ELISA)

Commercial kits were obtained to detect the release of tumor necrosis factor –α (TNF-α) and CXC-motif chemokine ligand 1 (CXCL-1) (Abcam, USA). Samples were prepared by collecting and storing them in appropriate tubes or vials. Wells were coated by adding 100 μL of the antigen solution to each well of the microplate and incubating at 4°C overnight. After incubation, wells were then blocked with 200 μL blocking buffer. After incubation for 1 h, the blocking buffer was removed and the primary antibody was added for 1 h. After incubation, 100 μL of enzyme conjugate solution was added and cultured for 1 h. Then, the substrate was added and cultured for 30 min in the dark. The absorbance value at a wavelength of 450 nanometers was determined using a plate reader within 30 min for computation.

### Western blot assay

Protein was extracted using radioimmunoprecipitation assay (RIPA) cell lysis buffer, and protein concentration was measured by bicinchoninic acid (BCA) assay. Sodium dodecyl sulfate-polyacrylamide gel electrophoresis (SDS-PAGE) was utilized to separate proteins, which were then moved to polyvinylidene fluoride (PVDF) membranes. The membranes were blocked for 60 min, followed by overnight incubation with primary antibodies against Acetyl-p53 (1:500, Beyotime, China), p21 (1:1000), SIRT3 (1:800), and β-actin (1:2000, Abcam, USA). The membranes were cultured with secondary antibodies (1:4000, Abcam, USA) for 1 h. Finally, enhanced chemiluminescence ECL developing solution was added evenly onto the membranes for exposure and development using Image Lab software for band analysis. Briefly, the background was subtracted, target bands were selected and the integrated optical density of the band was calculated to index protein concentration.

### Senescence-associated β-galactosidase (SA-β-gal) staining

The culture medium was aspirated, and 1.5 mL fixative was added for 6 min. For staining, the mixture was prepared according to the instructions: 1 mL staining solution (10×), 125 μL staining solution B, 125 μL staining solution C, 250 μL X-gal solution, and 8.5 mL ultra-pure water. Then, the fixative was aspirated, and 1 mL Phosphate buffer saline (PBS) was added to each well slowly to wash cells. Subsequently, 1 mL SA-β-gal staining mixture was added and cultured at 37°C overnight. Cells were observed and counted under an optical microscope (KEYENCE, Japan), and the percentage of senescent cells, that were positively stained with β-galactosidase, was calculated.

### Determination of telomerase activity

1 μl of cell lysate was added to the PCR tube containing the reaction mixture, and 1 μl of primer and Tag enzyme were added to the tube. Then, one drop of paraffin oil was added. After centrifugation for a few seconds, the tube was incubated at 30°C for 30 min. Then, PCR amplification was performed: 35 cycles were conducted at 94°C for 40 s, 50°C for 40 s, and 72°C for 60 s. Finally, the tube was incubated at 72°C for 5 min. The PCR products were electrophoresed on a 12.5% polyacrylamide gel and the expression of telomerase activity was determined by the detection of the 6 bp telomere DNA fragment product synthesized by telomerase [16].

### Statistical analysis

Statistical analysis was performed using SPSS 17.0 software. Data were expressed as (x ± s). Multiple comparisons were made using the one-way or two-way analysis of variance (ANOVA) followed by Tukey’s post-hoc test. *P* < 0.05 was considered statistically significant.

### Data availability

The data are available upon reasonable request from the corresponding author.

## RESULTS

### Orexin-2R was downregulated in IL-1β-cultured TC-28a2 chondrocytes

**Figure 1:**
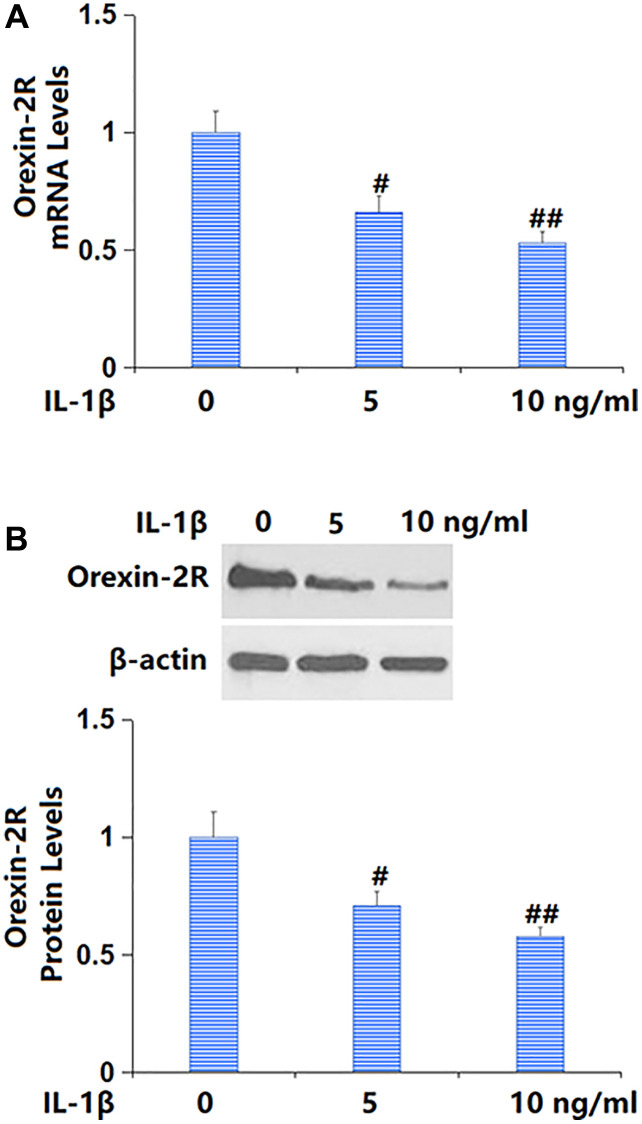
**Exposure to IL-1β reduced the expression of orexin-2 receptor (orexin-2R) in TC-28a2 chondrocytes in a dose-dependent manner.** Cells were stimulated with IL-1β (5, 10 ng/ml) for 24 hours. (**A**) mRNA levels of orexin-2R; (**B**) Protein levels of orexin-2R (^#, ##^*P* < 0.05, 0.01 vs. vehicle group, *n* = 6).

To predict the potential role of orexin-A, TC-28a2 chondrocytes were cultured with IL-1β (5, 10 ng/ml) for 24 h, followed by determining the change of orexin-2R level. The orexin-2R level was sharply repressed by 5 and 10 ng/ml IL-1β (Figure 1A, 1B), implying the possible protective role of orexin-2R/orexin-A in IL-1β-cultured TC-28a2 chondrocytes.

### Treatment with orexin-A inhibited IL-1β-triggered release of pro-inflammatory cytokines

**Figure 2:**
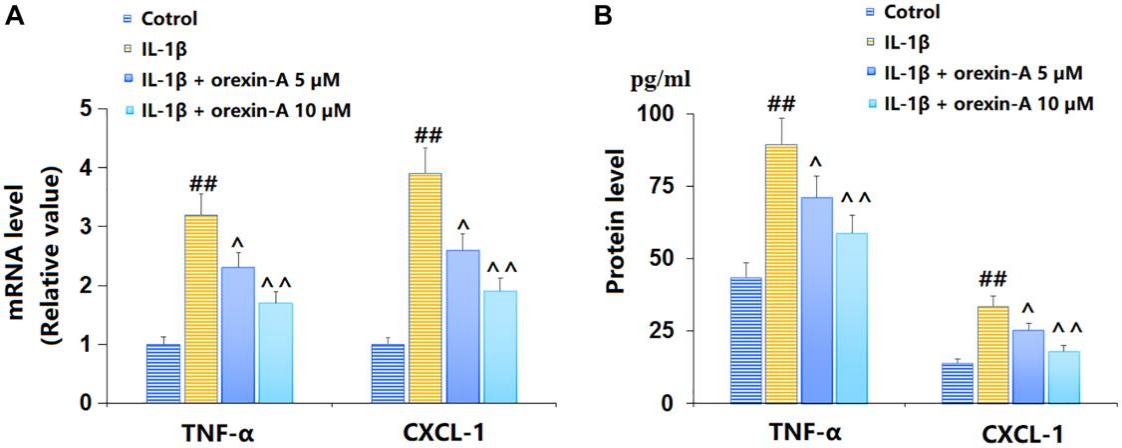
**Treatment with orexin-A inhibited IL-1β-induced expression of pro-inflammatory cytokines.** Cells were stimulated with IL-1β (10 ng/ml) in the presence or absence of orexin-A (5 and 10 μM) for 12 hours. (**A**) mRNA of TNF-α and CXCL-1; (**B**) Protein levels of TNF-α and CXCL-1 (^##^*P* < 0.01 vs. vehicle group; ^^, ^^^*P* < 0.05, 0.01 vs. IL-1β group, *n* = 6).

TC-28a2 chondrocytes were stimulated with IL-1β (10 ng/ml) with or without orexin-A (5 and 10 μM) for 12 h. The TNF-α and CXCL-1 mRNA levels were markedly elevated by IL-1β, but remarkably reduced by 5 and 10 μM orexin-A (Figure 2A). The TNF-α content released by chondrocytes was increased from 43.5 to 89.5 pg/ml by IL-1β, then largely repressed to 71.2 and 58.7 pg/ml by 5 and 10 μM orexin-A, respectively. Moreover, the CXCL-1 content in the control, IL-1β, 5 μM orexin-A, and 10 μM orexin-A groups was 13.9, 33.6, 25.2, and 18.1 pg/ml, respectively (Figure 2B).

### Treatment with orexin-A ameliorated IL-1β-triggered reduction in telomerase activity

**Figure 3:**
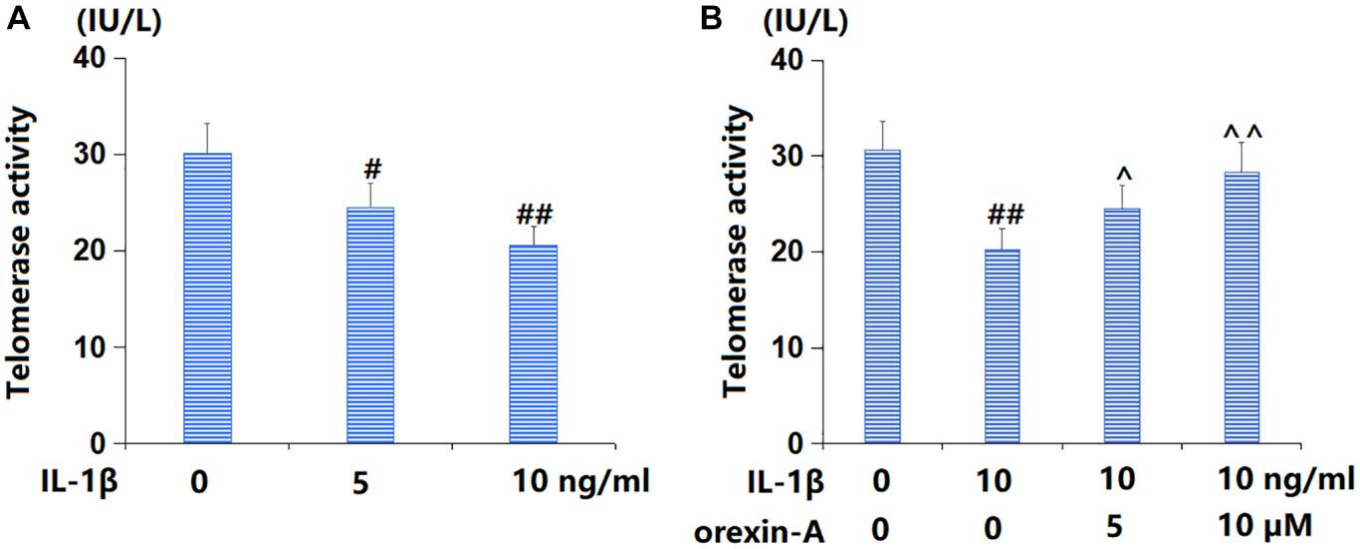
**Treatment with orexin-A ameliorated IL-1β-induced reduction in telomerase activity.** (**A**) Cells were stimulated with IL-1β (5, 10 ng/ml) for 14 days. Telomerase activity was measured; (**B**) Cells were stimulated with IL-1β (10 ng/ml) with or without orexin-A (5 and 10 μM) for 14 days. Telomerase activity was measured (^#, ##^*P* < 0.05, 0.01 vs. vehicle group; ^^, ^^^*P* < 0.05, 0.01 vs. IL-1β group, *n* = 5).

TC-28a2 chondrocytes were cultured with IL-1β (5, 10 ng/ml) for 14 d, followed by detecting the telomerase activity. The telomerase activity was distinctly reduced from 30.1 to 24.5 and 20.6 IU/L by 5 and 10 ng/ml IL-1β, respectively (Figure 3A). Subsequently, TC-28a2 chondrocytes were stimulated with 10 ng/ml IL-1β with or without orexin-A (5 and 10 μM) for 14 d. The telomerase activity was noticeably declined from 30.6 to 20.3 IU/L by IL-1β, then markedly elevated to 24.5 and 28.3 IU/L by 5 and 10 μM orexin-A, respectively (Figure 3B).

### Treatment with orexin-A ameliorated IL-1β-induced cellular senescence

**Figure 4:**
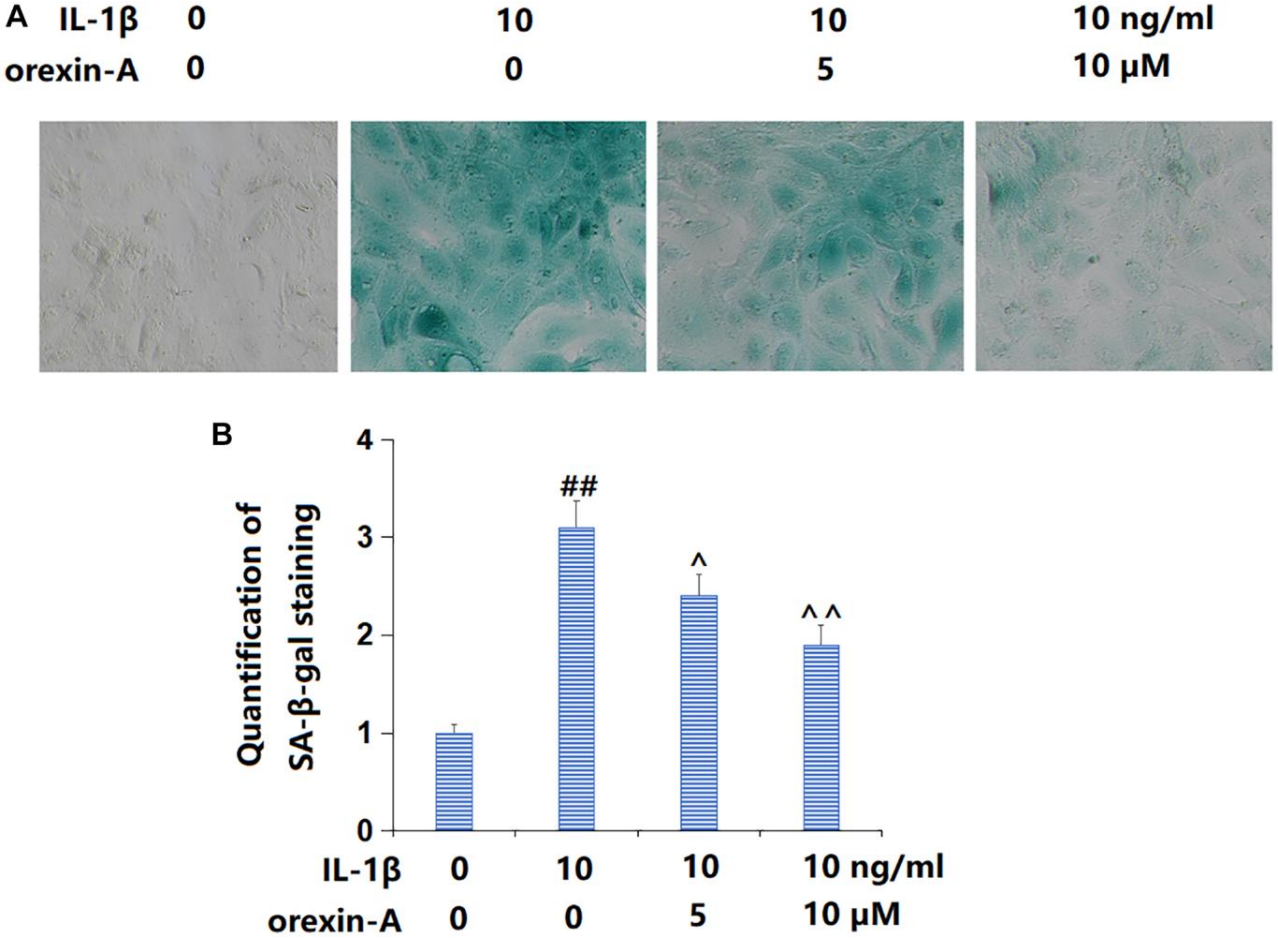
**Treatment with orexin-A ameliorated IL-1β-induced cellular senescence.** Cells were stimulated with IL-1β (10 ng/ml) with or without orexin-A (5 and 10 μM) for 14 days, cellular senescence was measured using senescence-associated β-galactosidase (SA-β-gal) staining. (**A**) Representative images of SA-β-gal staining; (**B**) Quantification of SA-β-gal staining (^##^*P* < 0.01 vs. vehicle group; ^^, ^^^*P* < 0.05, 0.01 vs. IL-1β group, *n* = 5).

Subsequently, the state of cellular senescence was checked. The percentage of SA-β-gal positive cells was observably increased by IL-1β, but largely reduced by 5 and 10 μM orexin-A (Figure 4A, 4B), suggesting a repressive function of orexin-A on IL-1β- induced cellular senescence in chondrocytes.

### Treatment with orexin-A repressed IL-1β-triggered activation of the p53/p21 axis

**Figure 5:**
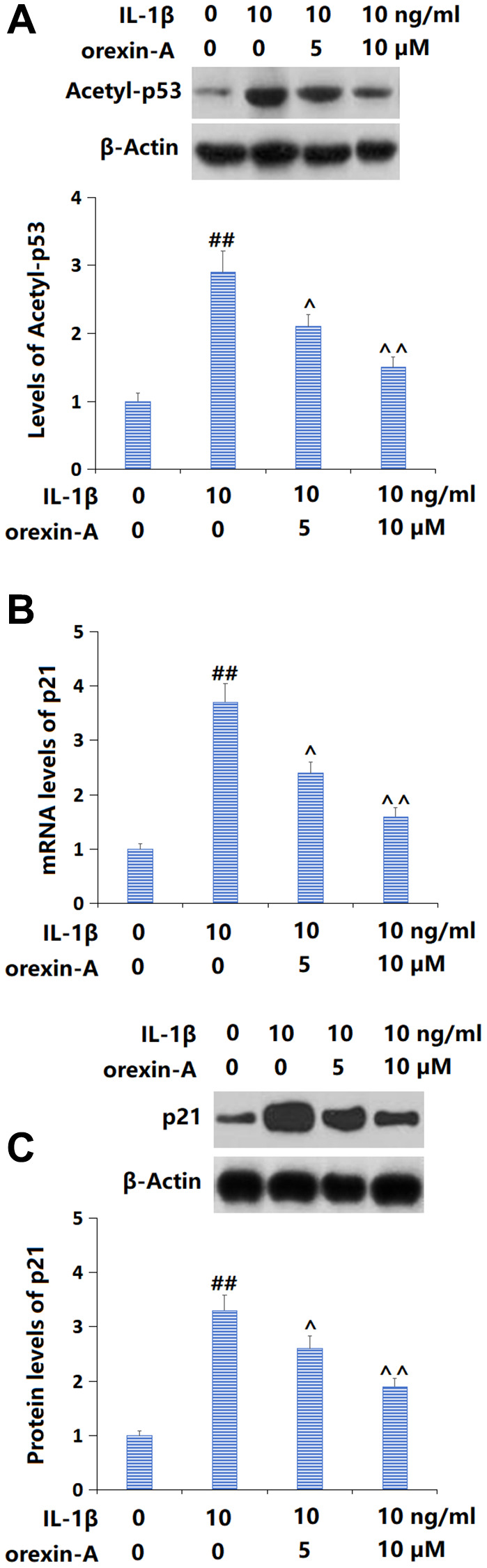
**Treatment with orexin-A prevented IL-1β-induced activation of the p53/p21 axis.** Cells were stimulated with IL-1β (10 ng/ml) with or without orexin-A (5 and 10 μM). (**A**) The levels of Acetyl-p53; (**B**) The mRNA levels of p21; (**C**) The protein levels of p21 (^##^*P* < 0.01 vs. vehicle group; ^^, ^^^*P* < 0.05, 0.01 vs. IL-1β group, *n* = 5).

p53/p21 signaling is a critical pathway involved in cellular senescence [17]. Levels of acetyl-p53 (Figure 5A) and p21 (Figure 5B, 5C) were sharply increased in IL-1β-cultured chondrocytes, but notably repressed by 5 and 10 μM orexin-A, implying a suppressive role of orexin-A against the activated p53/p21 axis in IL-1β-cultured chondrocytes.

### Treatment with orexin-A reversed IL-1β-triggered reduction of SIRT3

**Figure 6:**
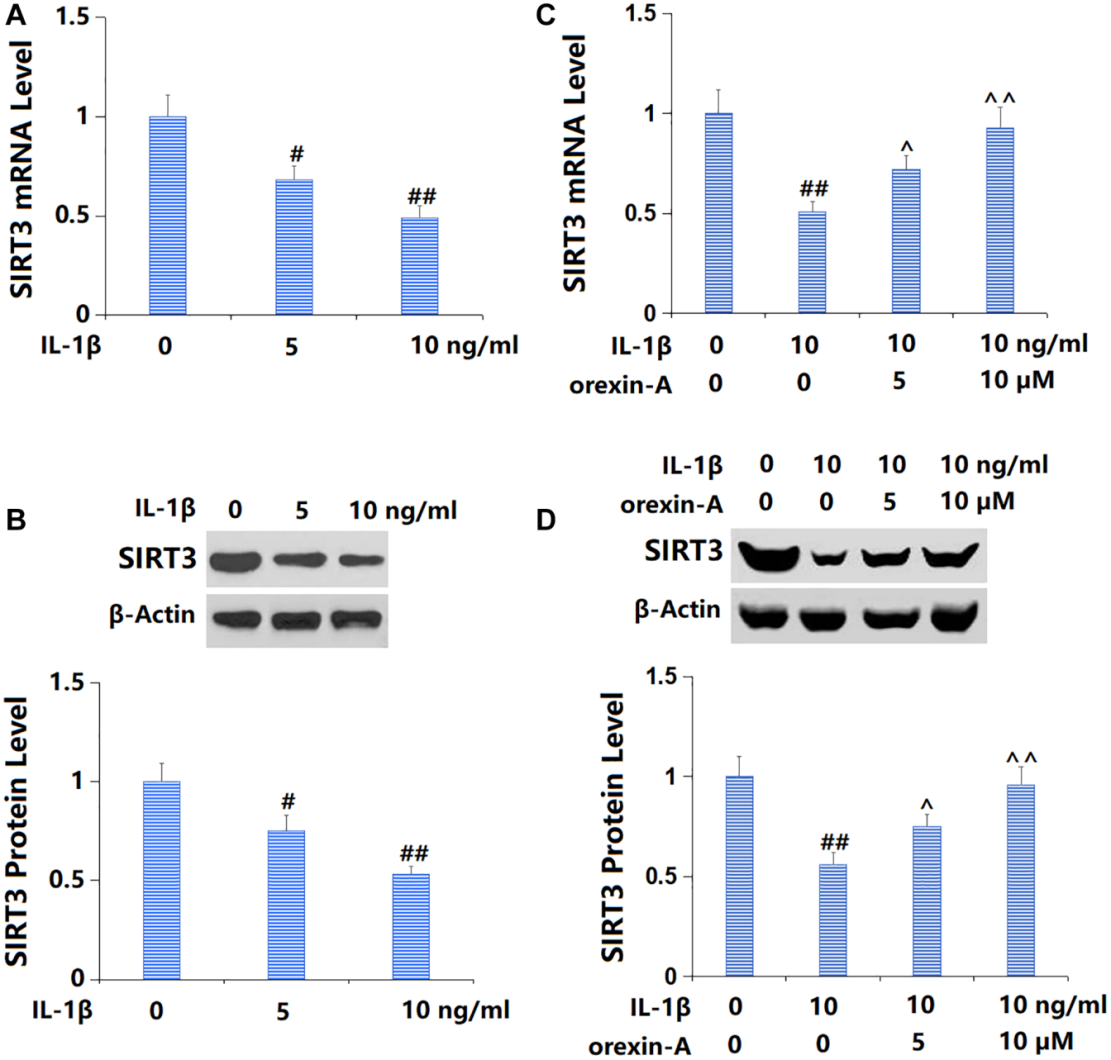
**Treatment with orexin-A attenuated IL-1β-induced reduction of SIRT3.** (**A**, **B**) Cells were stimulated with IL-1β (5, 10 ng/ml). mRNA and protein levels of SIRT3 were measured. (**C**, **D**) Cells were stimulated with IL-1β (10 ng/ml) with or without orexin-A (5 and 10 μM). mRNA and protein levels of SIRT3 were measured (^#, ##^*P* < 0.05, 0.01 vs. vehicle group; ^^, ^^^*P* < 0.05, 0.01 vs. IL-1β group, *n* = 5).

Firstly, TC-28a2 chondrocytes were cultured with IL-1β (5, 10 ng/ml) and SIRT3 was found remarkably downregulated (Figure 6A, 6B). Then, chondrocytes were stimulated with 10 ng/ml IL-1β with or without orexin-A (5 and 10 μM). The declined SIRT3 level observed in IL-1β-cultured chondrocytes was remarkably increased by 5 and 10 μM orexin-A (Figure 6C, 6D).

### Silencing of SIRT3 abolished beneficial effects of orexin-A in preventing cellular senescence

**Figure 7:**
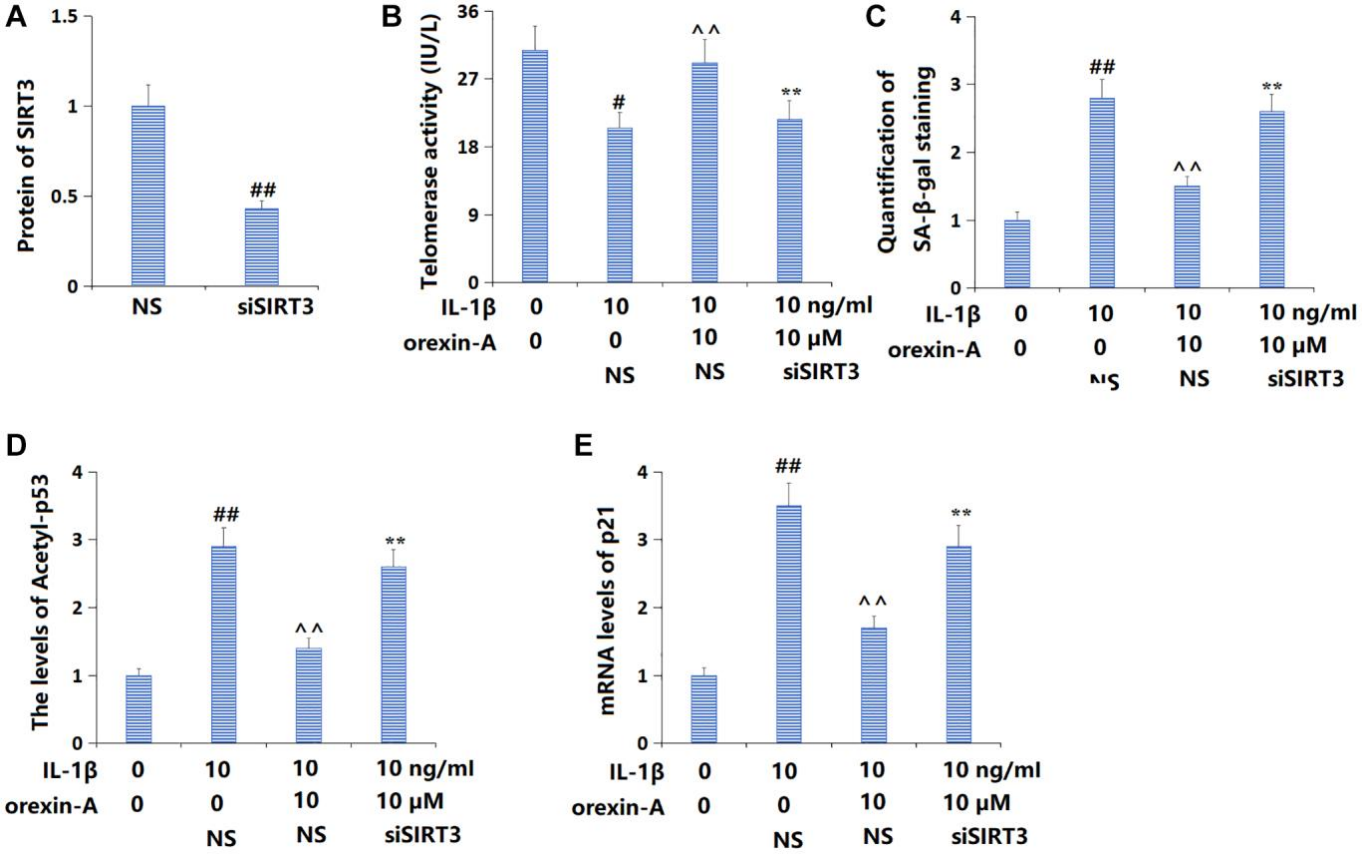
**Silencing of SIRT3 abolished the beneficial effects of orexin-A in preventing cellular senescence.** Cells were transducted Ad-SIRT3 shRNA, followed by stimulation with IL-1β (10 ng/ml) with or without orexin-A (10 μM). (**A**) Successful knockdown of SIRT3; (**B**) Telomerase activity was measured; (**C**) Quantification of SA-β-gal staining; (**D**) The levels of Acetyl-p53; (**E**) The mRNA levels of p21 (^##^*P* < 0.01 vs. vehicle group; ^^^^*P* < 0.01 vs. IL-1β group; ^**^P < 0.01 vs. IL-1β+ orexin-A group, *n* = 5).

To confirm the role of SIRT3 in the function of orexin-A, TC-28a2 chondrocytes were transduced with Ad-SIRT3 shRNA, followed by stimulation with IL-1β (10 ng/ml) with or without orexin-A (10 μM). The successful knockdown of SIRT3 in chondrocytes was confirmed by the Western blotting assay (Figure 7A). The telomerase activity was reduced from 30.8 to 20.5 IU/L by IL-1β, then elevated to 29.1 IU/L by orexin-A. Following the knockdown of SIRT3, the telomerase activity was reversed to 21.6 IU/L (Figure 7B). Furthermore, the increased proportion of SA-β-gal positive cells in IL-1β-cultured chondrocytes was markedly repressed by orexin-A but notably elevated by knocking down SIRT3 (Figure 7C). The upregulated acetyl-p53 (Figure 7D) and p21 (Figure 7E) in IL-1β-cultured chondrocytes were largely downregulated by orexin-A, but remarkably elevated by knocking down SIRT3.

## DISCUSSION

Cellular senescence is an irreversible cell cycle arrest that includes DNA damage, loss of mitochondrial function, activation of proto-oncogenes or tumor suppressor genes, and oxidative stress [18]. Characteristics of cellular senescence include increased SA-β-gal activity, upregulated senescence-associated proteins p53, p16, and p21, and elevated degradation of LaminB1 protein [19, 20]. OA involves multiple tissues throughout the joint, among which the degeneration and destruction of articular cartilage are most significant. Multiple cellular senescence markers, such as p16, p21, IL-6, and IL-8, have been identified in chondrocytes, chondroblasts, synovial fibroblasts, and infrapatellar fat pads of patients with OA [21]. Some studies have compared normal cartilage from the hip fracture of elderly patients with OA cartilage removed during joint arthroplasty and found β-gal staining only in OA chondrocytes. A previous study identified evidence of chondrocyte senescence in OA cartilage, including telomere length reduction and DNA damage, as well as increased p16 expression [22]. Senescent chondrocytes are found in articular cartilage following total knee arthroplasty and accumulate with age, and more senescent chondrocytes are observed in OA cartilage compared to age-matched healthy cartilage [22]. It has been found that senescent cells are only present in the diseased areas of OA joints, further confirming the relationship between OA and chondrocyte senescence [23]. Chondrocytes rarely proliferate under normal circumstances [24], suggesting that the presence of senescent cells in OA joints may be related to senescence processes rather than replicative senescence. Senescent cells secrete senescence-associated secretory phenotype (SASP) factors, such as matrix metalloproteinase (MMP)-1, MMP-3, and MMP-13 [25], leading to an imbalance between cartilage synthesis and degradation homeostasis, resulting in structural disorders and dysfunction [26]. Herein, in line with previous data reported by Huang [27] and Yin [28], declined telomerase activity and enhanced SA-β-gal activity were observed in IL-1β-cultured chondrocytes, accompanied by aggravated inflammation. Following orexin-A incubation, the telomerase and SA-β-gal activity were reversed, revealing a repressive function of orexin-A against chondrocyte senescence. Moreover, the p53/p21 axis was found activated in IL-1β-cultured chondrocytes, similar to the results presented by Shao [29]. The introduction of orexin-A notably repressed the p53/p21 axis, further confirming the anti-senescent activity of orexin-A.

SIRT3 is a member of the silent information regulator 2 protein (Sirtuin) family and a NAD-dependent deacetylase [30], which is primarily located in mitochondrial and nuclear fractions and is evidenced to be closely related to the human lifespan [31]. SIRT3 has been shown to regulate various biological processes of mitochondria, including ROS degradation, ATP production, mitochondrial dynamics, oxidation of nutrients, and mitochondrial unfolded protein response (UPR) [32]. Additionally, SIRT3 plays a key role in mitochondrial homeostasis, oxidative stress, metabolism, and genomic stability through its deacetylation function [33–35]. In recent years, multiple studies have demonstrated that SIRT3 significantly inhibits the process of cell senescence [35–37]. Herein, a downregulation of SIRT3 was observed in IL-1β-cultured chondrocytes, consistent with the research conducted by Wang [38]. The introduction of orexin-A largely increased the SIRT3 level, implying that the anti-senescent activity of orexin-A is possibly correlated to the SIRT3 activation. Furthermore, the impact of orexin-A on the cell senescence and p53/p21 axis in IL-1β-cultured chondrocytes was abrogated by silencing SIRT3, further confirming that SIRT3 participated in the anti-senescent activity of orexin-A. Both orexin-A and orexin-B are derived from a common precursor secreted by hypothalamic neurons. Different biological functions of orexin-A and orexin-B have been reported in previous studies. It should be noted that the potential benefits of orexin-B in OA are less reported. We will investigate whether orexin-B exerts anti-OA effects in our future studies.

In summary, orexin-A alleviated cell senescence in IL-1β-cultured chondrocytes by activating SIRT3. These findings suggest the novel potential for OA interventions by using orexin-A.
